# Supplementary material for: Reconciling Mining with the Conservation of Cave Biodiversity: A Quantitative Baseline to Help Establish Conservation Priorities
Source: PLoS One. 2016 Dec 20;11(12):e0168348. doi: 10.1371/journal.pone.0168348 (PMC5173368; doi:10.1371/journal.pone.0168348)
Supplement: S1 Dataset — (ZIP) [file pone.0168348.s002.zip › Taxa/Serra Norte/SN_2013/N4WS/Lista N4WS-24.pdf]

CAVIDADE N4WS-0024

Classe	Ordem	Fam/Outros	Gên/Outros	Espécie	S-U	U-S	Zon.
Arachnida	Araneae	Ctenidae		jovem	1		E
Arachnida	Araneae	Filistatidae		sp.1		X	E
Arachnida	Araneae	Mysmenidae		jovem	X		E
Arachnida	Araneae	Ochyroceratidae		jovem		X	E
Arachnida	Araneae	Pholcidae	*Mesabolivar*	*eberhard*	X		E
Arachnida	Araneae	Pholcidae	Ninetinae	sp.1	X		E
Arachnida	Araneae	Pholcidae		jovem	X	X	E/P
Arachnida	Araneae	Salticidae		sp.5	X	X	E
Arachnida	Araneae	Theridiosomatidae	*Plato*	sp.1	X		E
Arachnida	Araneae	Uloboridae	*Uloborus*	sp.1	X		E
Arachnida	Ricinulei	Ricinoididae	*Cryptocellus*	*tarsilae*		X	E
Entognatha	Collembola	Paronellidae		sp.4	X		E
Entognatha	Diplura	Campodeidae		sp.1		X	E
Insecta	Blattodea			jovem	1		E
Insecta	Diptera	Cecidomyiidae	Cecidomyiinae	sp.	X		E/P
Insecta	Diptera	Ceratopogonidae		sp.	X		P
Insecta	Diptera	Limoniidae		sp.	X	X	E
Insecta	Diptera	Tipulidae	Tipulinae	sp.	X		E
Insecta	Homoptera	Cixiidae		sp.3		X	E
Insecta	Hymenoptera	Chalcidoidea		sp.3		X	E
Insecta	Hymenoptera	Formicidae	*Camponotus*	sp.1	X		P
Insecta	Hymenoptera	Formicidae	*Gnamptogenys*	*striatula*		X	E
Insecta	Hymenoptera	Formicidae	*Pachycondyla*	*striata*		2	E
Insecta	Isoptera	Termitidae	*Atlantitermes*	sp.		X	E
Insecta	Isoptera	Termitidae	*Nasutitermes*	sp.	X		P
Insecta	Lepidoptera	Gelechioidea		sp.1		X	E
Insecta	Lepidoptera	Noctuoidea		sp.1	1		E
Insecta	Lepidoptera	Noctuoidea		sp.13	X		E
Insecta	Lepidoptera	Noctuoidea		sp.8		X	E
Insecta	Orthoptera	Phalangopsidae	*Paraclodes*	sp.	1	1	E
Insecta	Orthoptera	Phalangopsidae	*Phalangopsis*	sp.	2		P
Insecta	Psocoptera	Ptiloneuridae	*Ptiloneura*	sp.1		X	E
Symphyla		Scutigerellidae	*Hanseniella*	sp.1		X	E
Amphibia	Anura	Strabomantidae	*Pristimantis*	cf. *fenestratus*	2		E/P
Mammalia	Chiroptera	Emballonuridae	*Peropteryx*	sp.	1		E
